# Barriers and facilitators for engaging underrepresented ethnic minority populations in healthcare research: an umbrella review

**DOI:** 10.1186/s12939-025-02431-4

**Published:** 2025-03-12

**Authors:** Shahina Pardhan, Tarnjit Sehmbi, Rumalie Wijewickrama, Hugo Onumajuru, Mapa Prabhath Piyasena

**Affiliations:** Vision and Eye Research Institute, Anglia Ruskin University, East Rd, CB1 1PT Cambridge, UK

**Keywords:** Ethnic minorities, Racial minorities, Ethnic groups, Research, Research participation, Barriers, Obstacles, Challenges, UK

## Abstract

**Background:**

Research highlights that participation of ethnic minority individuals in research is low when compared to white counterparts. This poses challenges for healthcare planning and delivery, as lack of representativeness in research means that findings are generalised across all ethnic groups, and do not provide stakeholders with a full picture of how minority populations are affected. This contributes to health inequalities as these populations may then be underserved and not get the best possible management if differences due to ethnicity were to exist. This study synthesises the barriers to engaging minority individuals in research to understand, and enablers to better engagement of different minority communities in healthcare research.

**Methods:**

Five databases were searched (MEDLINE, CINAHL, PsycINFO and Web of Science and EMBASE) up to 29th April 2024, resulting in 897 articles, of which 11 met the inclusion criteria. Data were extracted from reviews and synthesised using qualitative meta-aggregation techniques. The socio-ecological framework was applied to synthesise the main outcomes. A protocol for this review was registered on PROSPERO (CRD42024532686).

**Results:**

The main barriers for research participation included: mistrust of healthcare professionals, research and researchers; socioeconomic and logistical challenges; language and cultural barriers; lack of awareness; external influences and perceived bias. Facilitators to support better research participation included: Community engagement and personalised approaches; culturally sensitive research strategies; linguistically appropriate study materials and study advertising; education workshops.

**Conclusions:**

To enable wider participation, it is important to understand not only the barriers but also to employ culturally appropriate facilitators, engaging with patient and public involvement (PPI) groups that communities trust, offer cultural training for researchers, and adopt a more collaborative and transparent way of working. This overview highlights the work that needs to be done on an intrapersonal, interpersonal, community and policy level to make research accessible and inclusive for ethnic minority groups.

## Introduction

While theory and principles on how to recruit ethnic minority groups into trials are accumulating [1], reported experiences and successful principles implemented into practice successfully are rare. The need to include ethnic minority groups in research is increasingly seen as important on scientific, policy, and ethical grounds [2]. The underrepresentation of minority groups in health research impacts many domains, such as the validity and generalisability of data [3], the development of services and interventions that meet their needs [4], resource allocation [5], and health inequalities which are perpetuated as a result of the omission of ethnic minority groups in research [6, 7].

Engagement refers to involvement, participation and active interest of groups in a particular activity [8]. Engagement of ethnic minority populations in research is essential for ensuring that scientific findings are representative and applicable to diverse communities [9]. Multifactorial reasons of underrepresentation via lack of engagement include lack of diversity inclusion in the overarching design of the study, assumptions on the part of researchers, and ethical procedures [10]. Typically, reasons for the underrepresentation have been directed towards the participants, for example reasons for non-participation have pointed towards failure of understanding or language barriers [9]. However, the lack of representation is more likely due to an array of complex factors that are multifaceted which this umbrella review aims to bring together.

The UK has long-standing ethnic variations in the prevalence of some diseases and in health outcomes [11], which have informed the need for ethnic classifications to be embedded in some health intervention guidelines (e.g. National Institute for Health and Care Research; NIHR). Research has shown there is insufficient participation and therefore underrepresentation of ethnic minority populations in research such as controlled trials and cohort studies in the UK and other parts of Europe, and in the USA [12–15]. It is accepted that the inclusion in medical research of people from Black, Asian and Minority Ethnic (BAME) groups is necessary to avoid unwarranted inequalities, and to guard against an under representative healthcare evidence base [12]. There is, nevertheless, strong evidence to suggest that people from BAME groups are underrepresented in various UK medical research contexts [16].

In some parts of the world, ethnic minority inclusion in research has become a standard part of research practice. For example. in the USA, the National Institute of Health (NIH) regulation mandates the inclusion of ethnic minority groups in clinical trials. Hence, around 30% of participants in clinical research are from ethnic minority groups and that has remained stable since the regulation was introduced. Conversely, whilst there are several guidelines and frameworks aimed at encouraging diversity and promoting inclusivity in health research, in the UK there is no such mandating regulation for clinical researchers [17]. This is reflected in the underrepresentation of minority groups in research in the UK [18]. This underrepresentation of ethnic minority groups in research contributes to inequalities in healthcare services [9]. A survey carried out by the National Institute of Health Research (NIHR) reported that half that of the recruiting studies (64%) completely excluded participants who were unable to communicate in English [17], highlighting the extent of the issue. The underrepresentation of ethnic minorities has significant ethical implications, as research findings are not representative of ethnic minority groups and therefore have limited generalisability [4].

As well as ethical implications, there are several other implications of the underrepresentation of diverse groups in health research on healthcare outcomes. For example, underrepresentation in clinical research can contribute to significant health disparities, such as treatments and interventions being tailored to the majority population [2, 3]. These inequalities can result in poorer health outcomes for underrepresented groups. The lack of diversity in clinical research can also result in biased data that fails to reflect the needs of ethnic minority groups [6, 7]. Hence, healthcare policies and interventions may not be suitable to address the needs of underrepresented groups effectively. Additionally, underrepresentation can impact healthcare access and hence result in poorer health outcomes in these populations [7]. Ultimately, this underrepresentation in research is detrimental and can have profound implications for health equity. Ensuring that ethnic minority populations are represented in healthcare research is critical for improving healthcare outcomes, reducing disparities, and building personalised care.

This review aims to synthesise the research on barriers and facilitators for ethnic minority research engagement in different areas of health research. The socio-ecological framework has been applied in this review due to it offering a comprehensive, multi-dimensional approach to understanding barriers and facilitators for engaging underrepresented ethnic minority populations in healthcare research. By considering several levels of influence, the complexity of the issue can be captured, to aid the identification of appropriate facilitators. This framework is valuable in identifying actionable strategies for reducing disparities and promoting inclusivity in research. We aim to adapt and learn from studies conducted in high income countries, with insight from UK based review groups and collaborators to ensure applicability for UK based research. Understanding barriers and facilitators for research engagement is crucial for developing strategies to improve inclusivity and the validity of research findings. This umbrella review aims to build on previous reviews and bring these factors to light in order for researchers to develop inclusive strategies to improve participation into research by ethnic minority groups. This has been done in line with a socio-ecological framework with an aim to outline actionable facilitators for researchers.

## Materials and methods

### Search strategy and selection criteria

Five databases were searched for reviews such as systematic reviews and scoping reviews: PsycINFO (Ovid), MEDLINE (Ovid), and CINAHL PLUS (EBSCO), Web of Science, EMBASE. The final search was carried out on 29 April 2024. No restrictions were placed on publication period, however only studies published in English have been included. Ethnic minority populations will be referred to as all ethnic groups apart from the White British group [19]. Searches were conducted using keywords connected with Boolean terms to maximise the search. Reference lists of selected full reports were also be searched to ensure all relevant studies were identified through this process.

We explored (i) teachings from the current literature around the barriers and (ii) recommendations from these studies as facilitators. The method started with a general query on the key barriers and facilitators for ethnic minority populations when engaging in research and consisted of conducting a search using key words (see Table 1).

**Table 1:** Keywords and boolean terms used in EBSCO

Databases	Keywords
PsycInfo (424), CINAHL (224), MEDLINE (688)	Ethnic minorities *OR* Racial minorities *OR* Ethnic groups *AND* Research *OR* “Research participation” OR “RESEARCH ENGAGEMENT” *AND* Barriers *OR* Obstacles *OR* Challenges *AND* UK *OR* “United Kingdom” OR ENGLAND OR BRITAIN OR WALES OR SCOTLAND OR “NORTHEN IRELAND” AND REVIEW* OR “META ANALYSIS” OR “SYSTEMATIC REVIEW”

Inclusion criteria were peer reviewed and published reviews. This could be systematic reviews, meta-analyses, literature reviews, narrative reviews, rapid reviews or scoping reviews. Only reviews conducted by UK authors or in collaboration with UK authors were included in this review. The review aimed to gather and synthesise the reflections of expert reviewers in the UK on this topic. Other high income and low-income country studies with non-UK based reviewers were excluded. This was done to maximise applicability of review findings to the UK healthcare system. Healthcare research is systemically varied in different countries. Additionally, non-UK studies may be influenced by policies that are not applicable or comparable in the UK context. To provide in depth insight into the topic for UK based researchers, non-UK based research was excluded. Full reports which are not reviews, and grey literature were excluded. No restrictions were placed on year of publication.

**Fig. 1:**
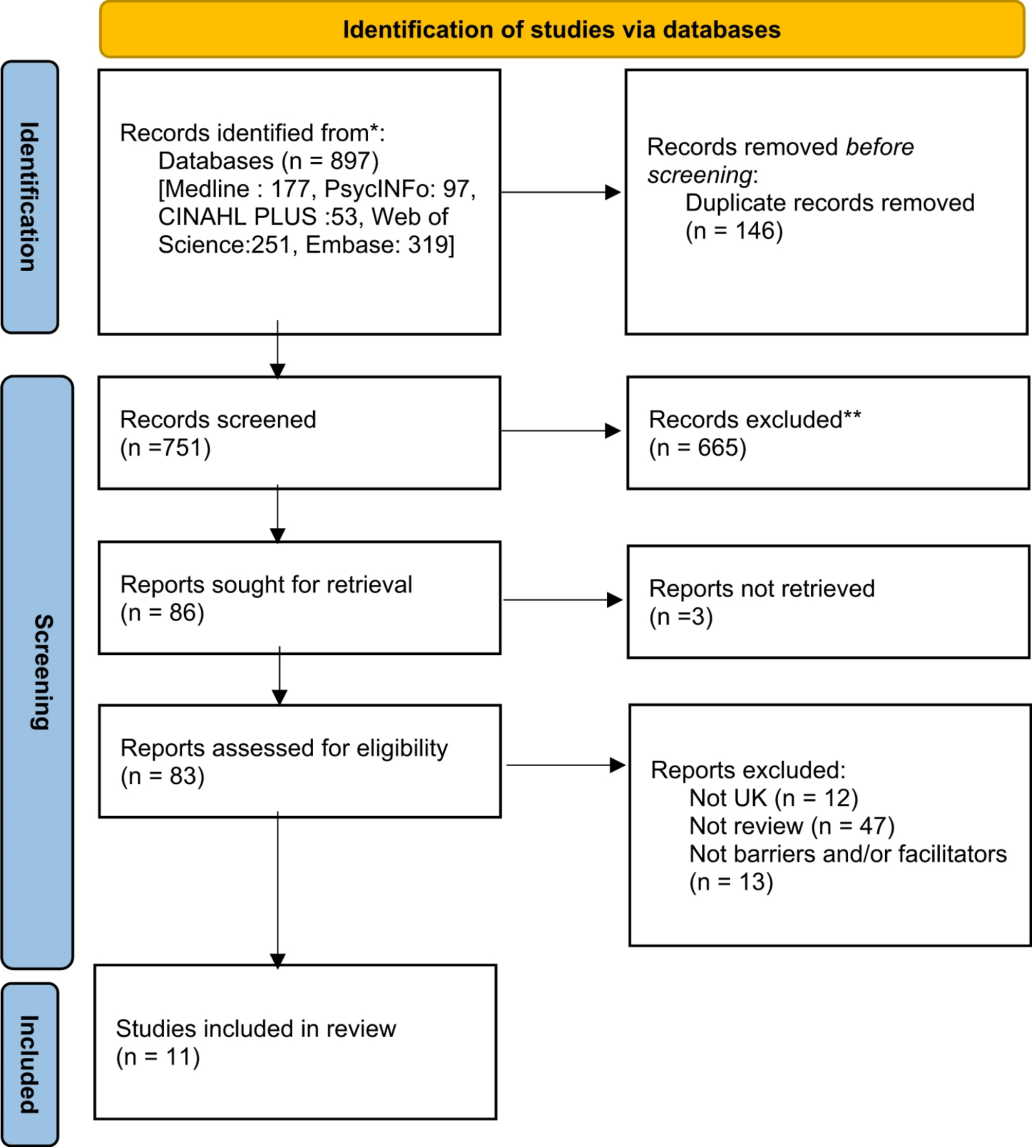
PRISMA flow chart

**Table 2:** Risk of Bias using JBI critical appraisal checklist for systematic reviews and research syntheses

JBI Critical Appraisal Checklist for Systematic Reviews and Research Syntheses												
	1. Is the review question clearly and explicitly stated?	2. Were the inclusion criteria appropriate for the review question?	3. Was the search strategy appropriate?	4. Were the sources and resources used to search for studies adequate?	5. Were the criteria for appraising studies appropriate?	6. Was critical appraisal conducted by two or more reviewers independently?	7. Were there methods to minimize errors in data extraction?	8. Were the methods used to combine studies appropriate?	9. Was the likelihood of publication bias assessed?	10. Were recommendations for policy and/or practice supported by the reported data?	11. Were the specific directives for new research appropriate?	Overall appraisal
Brown et al. (2014)	Y	Y	Y	Y	Y	Y	Y	Y	Y	Y	Y	Include
Waheed et al. (2020)	Y	Y	Y	Y	Y	Y	Y	Y	Y	Y	Y	Include
Waheed et al. (2015)	Y	Y	Y	Y	Y	Y	Y	Y	N/A	Y	Y	Include
Woodal et al. (2010)	Y	Y	Y	Y	Y	Uncertain	Uncertain	Y	N	Y	Y	Include
Hussain-Gambles et al. (2004)	Y	Y	Y	Y	Y	Uncertain	Uncertain	Y	N	Y	Y	Include
Symonds et al. (2012)	Y	Y	Y	Y	Y	Y	Y	Y	N	Y	Y	Include
Liljas et al. (2017)	Y	Y	Y	Y	Y	Y	Y	Y	N	Y	Y	Include
Spencer et al. (2022)	Y	Y	Y	Y	Y	Y	Y	Y	N	Y	Y	Include
Bodicoat et al. (2021)	Y	Y	Y	Y	Y	Y	Y	Y	N	Y	Y	Include
Bonevski et al. (2014)	Y	Y	Y	Y	Y	Y	Y	Y	N	Y	Y	Include
Masood et al. (2019)	Y	Y	Y	Y	Y	Y	Y	Y	N	Y	Y	Include

### Selection of studies and data extraction

The databases were systematically searched in April 2024, with the final search being carried out on 29th April 2024. Duplicates were removed prior to the screening using RefWorks (See Fig. 1. for PRISMA flowchart). The author (TS) screened all studies based on titles, and then abstracts, and finally full-text reports. A second reviewer assisted with 100% of the title and screening stage (RW). The final selected papers were cross-checked by additional reviewers (HO, RW) at the full text report data extraction stage, and any conflicting views were resolved through discussion (TS, HO, RW). Eleven articles were considered eligible and were examined in full text. Study quality and risk of bias assessment was carried out by two reviewers (TS, RW). For each review, the author, year of publication, location, sample, and summary of key findings were extracted (TS) (See Table 3 for study table).

**Table 3:** Data extracted from key studies included in this review

Authors	Study title	Country review was conducted in	Number of studies included	Topic explored	Type of Study	Key Findings
Brown et al. 2014	Barriers to recruiting ethnic minorities to mental health research: A systematic review	UK	9	Mental health research	Systematic review	This review identified various key barriers to the recruitment of ethnic minorities to mental health research. Key findings include pervasive mistrust of research institutions due to historical and ongoing experiences of discrimination, which discourages participation. Cultural and linguistic differences were also highlighted as major barriers, with many studies noting the lack of culturally sensitive communication and materials. Socioeconomic factors, such as financial constraints and limited access to healthcare, further hinder participation. Additionally, the review found that research designs often fail to consider the specific needs and contexts of ethnic minority communities, leading to perceptions of irrelevance. The study concludes that overcoming these barriers requires a multifaceted approach, including building trust, improving cultural competence, and addressing systemic issues within research practices.
Waheed et al. 2020	Recruitment and methodological issues in conducting dementia research in British ethnic minorities: A qualitative systematic review.	UK	33	dementia research	Systematic review	This review aimed to explore and understand the challenges and barriers associated with recruiting British ethnic minority groups into dementia research. Language barriers and a lack of culturally appropriate research materials were also highlighted as major obstacles, complicating the informed consent process and participant understanding. The review also pointed to mistrust of research institutions, partly due to historical experiences of discrimination, as a barrier to participation. Additionally, the study identified methodological issues such as the lack of diversity among research staff and the failure to consider cultural nuances in study designs, which further impede effective recruitment and engagement. The review concludes that addressing these challenges requires culturally sensitive approaches, better community engagement, and more inclusive research practices tailored to the needs of ethnic minority populations.
Waheed et al. 2015	Overcoming barriers to recruiting ethnic minorities to mental health research: A typology of recruitment strategies.	UK	9	mental health research	Narrative review	The aim of this review was to identify and evaluate different strategies used to overcome barriers to recruiting ethnic minority populations into mental health research. he review identified key barriers such as mistrust in research institutions, cultural and linguistic differences, and logistical challenges. To address these, the study developed a typology of effective recruitment strategies, which included building trust through community engagement, employing culturally competent research staff, and using tailored communication methods. The key findings highlight the importance of culturally sensitive approaches and the need for personalized recruitment strategies to improve the inclusivity and representation of ethnic minorities in mental health research.
Woodal et al. 2010	Barriers to participation in mental health research: Are there specific gender, ethnicity and age related barriers?	UK	49	mental health research	Systematic Review	This review identified distinct challenges faced by different demographic groups in mental health research participation. Ethnicity-related barriers were found to significantly impact participation in mental health research. Key findings include a deep-seated mistrust of research institutions among ethnic minority groups, often rooted in historical experiences of exploitation and discrimination. Cultural stigmas surrounding mental health within these communities further discourage participation, as mental illness may be viewed as a taboo subject. Language barriers also pose a significant challenge, with many participants struggling to understand research materials and communication if not presented in their native language or in a culturally relevant manner. Additionally, the study found that research designs often fail to consider the specific cultural contexts of ethnic minorities, leading to perceptions of irrelevance or exclusion. Addressing these barriers requires culturally sensitive recruitment strategies and efforts to build trust within these communities.
Hussain-Gambles et al. 2004	Why ethnic minority groups are under-represented in clinical trials: a review of the literature.	UK	6	Clinical trials	Narrative review	This review highlights several key factors contributing to the underrepresentation of ethnic minority groups in clinical trials. Mistrust of medical research and healthcare institutions, stemming from historical and ongoing discrimination, is a significant barrier. Cultural and linguistic differences also play a crucial role, as many ethnic minority participants face challenges in understanding the purpose of trials or the implications of participation. The review identifies socioeconomic barriers, such as limited access to healthcare and financial constraints, which further restrict participation. Additionally, there is a lack of targeted outreach and recruitment strategies that consider the specific needs and concerns of these communities. The review concludes that to increase participation, clinical trials must implement culturally tailored approaches, build trust within minority communities, and address the systemic issues that contribute to their exclusion.
Symonds et al., 2012	Recruitment of ethnic minorities into cancer clinical trials: experience from the front lines	UK & US	33	Cancer clinical trial	Narrative review	This review explores the practical challenges and barriers faced by researchers in recruiting ethnic minority participants for cancer clinical trials. Key findings reveal that mistrust of medical research, often rooted in historical injustices, is a major obstacle to participation. Cultural differences and language barriers further complicate recruitment efforts, as potential participants may have difficulty understanding trial information or may be hesitant due to differing cultural perceptions of cancer and treatment. Additionally, logistical challenges such as transportation, time commitments, and financial constraints were identified as significant deterrents. The study emphasizes the need for culturally sensitive recruitment strategies, including the use of community outreach and education to build trust and improve understanding of clinical trials among ethnic minority groups.
Liljas et al., 2017	Strategies to improve engagement of ‘hard to reach’ older people in research on health promotion: a systematic review	UK	23	Health promotion research	Systematic review	This review identifies key strategies for successfully involving older adults, particularly those considered ‘hard to reach,’ in health promotion research. The review highlights that barriers such as social isolation, mistrust in research, and physical or cognitive impairments often hinder participation among older adults. Successful engagement strategies identified include building trust through community-based approaches, using personalized and face-to-face recruitment methods, and ensuring that communication is clear, accessible, and tailored to the needs of older individuals. Additionally, involving older adults in the research design process and offering flexible participation options, such as home visits or simplified procedures, were found to be effective. The review concludes that a combination of these tailored strategies is essential to improve the inclusion and engagement of older, hard-to-reach populations in health promotion research.
Spencer et al., 2022	A systematic review of the experiences of minority language users in health and social care research.	UK	74	Health and social care research	Systematic review	This systematic review discusses several critical challenges faced by minority language users in engaging with health and social care research. Key findings include significant barriers such as language difficulties, which hinder participants’ ability to understand research materials, consent forms, and communication with researchers. The review also highlights issues related to cultural differences and mistrust of research institutions, which can further impede participation. Effective strategies identified include employing bilingual research staff, providing translated materials, and adapting research methods to be more culturally sensitive. The review underscores the need for improved accessibility and inclusivity in research practices to better accommodate minority language users and ensure their equitable participation in health and social care research.
Bodicoat et al., 2021	Promoting inclusion in clinical trials-a rapid review of the literature and recommendations for action	UK	72	Improving participation of ethnic minorities in clinical trials.	Rapid review	This review identifies key barriers to the inclusion of diverse populations in clinical trials and offers actionable recommendations to enhance participation. Key findings include persistent issues such as socioeconomic barriers, cultural and linguistic differences, and historical mistrust of medical research among underrepresented groups. The review emphasizes the need for strategies that address these barriers, such as implementing community engagement initiatives, using culturally competent staff, and providing accessible information in multiple languages. Recommendations also highlight the importance of redesigning trial protocols to accommodate the specific needs of diverse populations, improving outreach efforts, and ensuring equitable access to clinical trials. These measures are essential to achieving more inclusive and representative clinical research.
Bonevski et al., 2014	Reaching the hard-to-reach: a systematic review of strategies for improving health and medical research with socially disadvantaged groups	UK & Australia	31	Improving health research participation among socially disadvantaged groups	Systematic review	This review highlights several effective strategies for enhancing research participation among socially disadvantaged populations. Key findings include the importance of employing community-based approaches to build trust and engage participants, using culturally and linguistically appropriate materials, and addressing logistical barriers such as transportation and financial constraints. The review also emphasizes the need for researchers to adopt flexible and adaptive recruitment methods, including offering incentives and utilizing outreach through trusted community organizations. By implementing these strategies, researchers can better reach and include socially disadvantaged groups, ensuring that health and medical research findings are more representative and equitable.
Masood et al., 2019	Synthesis of researcher reported strategies to recruit adults of ethnic minorities to clinical trials in the United Kingdom: A systematic review	UK	21	Identifying strategies for recruitment of minority groups in clinical trials	Systematic review	This review identifies key challenges and strategies in recruiting ethnic minorities to clinical trials. The review highlights barriers such as mistrust, language, and cultural differences, and emphasizes the importance of community engagement, culturally tailored materials, and diverse recruiters. Effective recruitment relies on trust-building, flexible trial designs, and early collaboration with minority communities. The paper calls for more standardized reporting and future research into subgroup-specific strategies to improve the inclusion of ethnic minorities in clinical research.

### Study quality appraisal

The Joanna Briggs Institute (JBI) Critical Appraisal Checklist for Systematic Reviews and Research Syntheses was used to assess the methodological quality of the reviews included in this umbrella review. This tool serves as a guide to assess the quality of the included studies using a checklist consisting of 11 questions (Q1–Q11). Each question has the option of yes, no, uncertain, or not applicable [20]. Reviews which were rated with fewer than five ‘yes’ responses were excluded. No reviews were excluded in the appraisal stage. The results of this evaluation indicate each review’s level of quality and are presented in Table 2. Any discrepancies during the quality appraisal were resolved through open discussion.

### Data synthesis

Data were extracted according to the review question, and the findings were synthesised to allow identification of the barriers and facilitators of engaging ethnic minority groups in research [21], as well as suggestions for strategies to engage minority populations in research.

The data were synthesised using the guidelines outlined by Aromataris and Pearson [22]. This consists of the JBI approach to qualitative synthesis, which suggests that the meta-aggregative approach is sensitive to the practicality and usability of research findings [22, 23], and was therefore adopted. The features of the meta-aggregative review included a clearly defined objective, detailed inclusion and exclusion criteria, a comprehensive research strategy, quality appraisal of included research, analysis of the data extracted, presentation and synthesis of the findings, and transparent reporting of the approach undertaken [24]. Categorisation involves repeated, detailed examination of the findings [24]. Findings were grouped in order to develop categories based on similarity in concepts. Category descriptions were created by consensus process between authors (TS, MP). Common barriers and facilitators identified across reviews were categorised in line with the conceptual framework of the socio-ecological model which contains 4 levels. The levels that make up the socio-economic framework are: interpersonal, intrapersonal, community and policy [25]. We aimed to capture the multifaceted nature of research participation, as the framework suggests identifying and targeting barriers and facilitators at multiple levels rather than a single level.

## Result

**Fig. 2:**
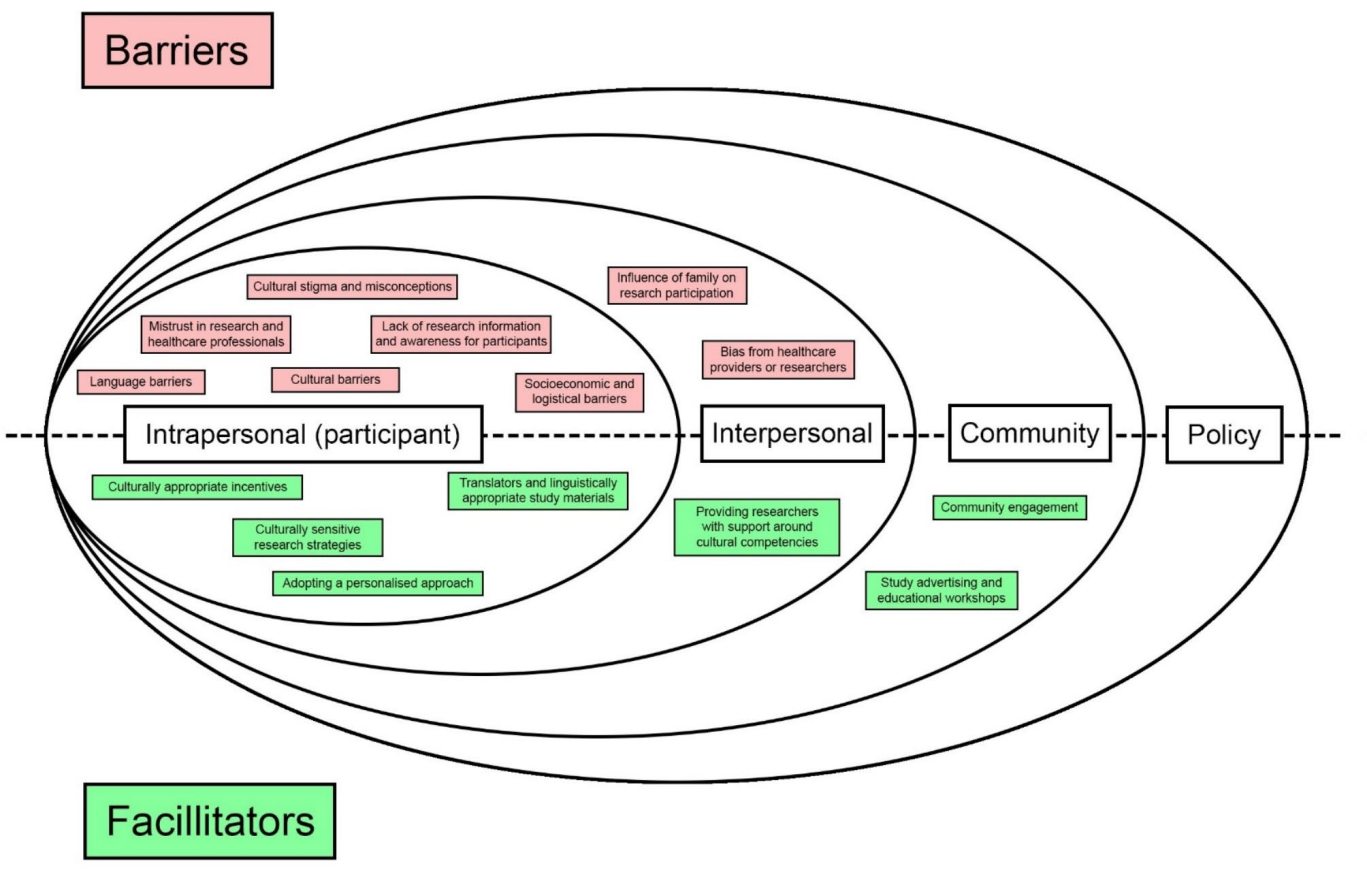
Key barriers and facilitators shown in line with the socio-ecological framework

Eleven reviews were identified which were relevant to barriers and facilitators to ethnic minority research participation. This umbrella review consists of seven systematic reviews [17, 18, 26–30], three narrative reviews [4, 31, 32] and a rapid review [33] and summarised in Table 3. All reviews explored the barriers and facilitators of engaging ethnic minority populations in healthcare research and made recommendations for future research based on their findings. The reviews explored an array of research topics: three studies explored participation in mental health research [18, 30, 32], one in dementia research [26], four reviews in participation in clinical trials [4, 17, 33], one in health and social care research [28], one in health promotion research [27] and one in health and medicine research [29]. A summary of the barriers and facilitators of research engagement in line with the socio-economic model is presented in Fig. 2.

### Barriers for engaging ethnic minority individuals in research

#### Language barriers

Language challenges were shown to be a significant barrier to research participation [4, 17, 18, 26, 27, 29–33]. Good communication and understanding are vital when providing potential participants with study information and hence recruitment. In addition to verbal communication, lack of translated materials was also shown as a barrier to participation as potential participants might not be able to access the research and understand the research commitments [27]. In addition, this lack of consideration for research accessibility for the diverse population may be perceived as disrespectful to ethnic minority groups.

In order to address this, participants would translate the assessment themselves, posing many challenges. Lack of standard guidelines on translating assessment and lack of standard translating versions would make this even more challenging and inaccurate.

The context of the study is also important. Some English words are difficult to translate into other languages, for example assessment questions exploring psychiatric or psychological symptoms are difficult to translate from English to South Asian languages due to the lack of necessary vocabulary in the language [26]. Language barriers have the greatest impact when obtaining consent from trial participants. The increasing complexity of consent forms and information sheets may confuse and cause potential participants to be fearful of the research [4]. Older individuals from ethnic minority backgrounds who rely on others for translation are also more likely to engage with others who speak their native language. If alongside language, participants are unfamiliar with the research process (which is also a barrier) this is likely to cause further confusion. Language issues may also pose difficulties for specific research, for example in dementia research, as it is difficult to assess cognition of participants with tests based on the English language if they cannot speak or understand English. Having validated assessment tools in South Asian languages such as Punjabi, Gujarati and Bengali would be useful [26].

#### Cultural barriers

In addition to translating the study materials into the required languages, it is important to ensure the information is culturally appropriate [17]. In translating written materials, there is a potential risk that some directly translated words may be perceived as stigmatising or confusing [18]. Input from a culturally competent advisor would be beneficial to ensure materials are culturally appropriate and not just language translations [18]. Studies emphasised the importance of having culturally appropriate interviews and scales with accurate translations [26]. Lack of fully validated culturally adapted assessments across ethnicities could act as a barrier to study participation [26]. Cultural appropriateness is likely to differ across context and ethnicities, therefore it is important to ensure that this is done on a case-by-case basis. For example, mental health research may hold more stigma and therefore require specific cultural sensitivity protocols compared to research exploring another health condition [18, 32].

Additionally, incentives may have a negative impact on recruitment [18]. Some Asian elders may see incentives as culturally inappropriate and not feel comfortable accepting monetary incentives, or feel information was being sold as part of a material exchange. Therefore, culturally inappropriate incentives may impact recruitment or retention rates, and make potential participants sceptical of the research and its’ goals [18]. For this reason, it is important to be transparent with participants with the goals of research and ensure cultural appropriateness is assessed at the research planning stage to prevent this becoming an issue.

#### Cultural stigma and misconceptions

Cultural stigma may be a barrier in some areas such mental health and psychotherapy [18, 32]. Potential participants may have concerns regarding the confidentiality of the research, which might be linked to a lack of understanding. The fear of being stigmatised as a result of study participation or being perceived as mentally ill is a potential barrier to recruitment [18, 30], especially in Asian communities, despite a limited exposure to research [18]. This may be due to the collectivist culture present in some Asian cultures, and concerns surrounding how a mental health diagnosis may impact the reputation of the family [18]. Additionally, gender can be a barrier for ethnic minority populations engaging in research due to a woman’s perceived ‘traditional’ role as a woman of the house, wife and mother, and research participation may be viewed as ‘selfish’ [18].

Stigma can act as a barrier in dementia research [26] with some ethnic minority communities perceiving a dementia diagnosis as unimportant as its symptoms considered a consequence of regular ageing [26]. Similarly, in clinical trials on topics such as cancer and the benefits of treatment, the associated stigma of the disease, for both the individuals and family, can be a barrier with regards to treatment and participation in trials [31]. This suggests that the context of the research can add to the stigma participants perceive.

Other barriers include beliefs among older adults that they are too old to participate, concerns about side effects or taking an experimental medicine, stress, conservatism attitude to risk taking, religious beliefs, ‘Guinea-pig’ perceptions, not feeling comfortable or respected, concerns around confidentiality, and negative attitudes to clinical trials. In addition, social approval is important including of family and communities, and lack of it may prevent participation [4, 27, 29]. This suggests the importance of raising awareness of the benefits of research participation in community groups.

#### Mistrust in research and healthcare providers

Mistrust in researchers and healthcare professionals has been reported as a barrier to participation for ethnic minority groups [17, 18, 32], possibly linked to legacy of unethical research conducted in the past, particularly the Tuskegee scandal which involved Black people not being offered efficacious syphilis treatment [18]. Another element of mistrust stems from participants suspicions about healthcare services [18, 34]. African women voiced suspicion about mental health services, were suspicious of physicians and compared them to policemen, perceiving encounters to be followed by hospitalisation [35], with other researchers reporting that individuals found it difficult trusting professionals they did not know [30] and viewed the consent process as suspicious [18]. Mistrust of research or researchers can lead to the perception that participation in research presents no personal benefit to the participants or their community, and may potentially cause harm, stigma, mistreatment or exploitation [29]. This barrier may also be related to the lack of awareness of what research participation involves in ethnic minority populations, as this lack of awareness can feed into these stereotypes. It is important to recognise these misconceptions and work towards creating honest and transparent research that is accessible for all populations.

#### Socioeconomic and logistical barriers

Socioeconomic factors may be a barrier to research participation, linked to costs of research participation, and lack of time due to work or family commitments [4, 17, 26, 31]. Logistical barriers which hindered participation included location of the study as non-familiar places may result in study withdrawal or reluctancy to participate. Lack of childcare or lack of transportation can also be a barrier to study participation. Although these barriers are not specific to ethnicity, research suggests that people of ethnic minority background from low-income areas expressed higher levels of reluctance to participate in research [18].

Lack of financial resources has been noted as a barrier for both participants and researchers [26]. Research conducted with the Afro-Caribbean community highlighted lack of funding for research with marginalised communities [26], suggesting that research with ethnic minority communities requires adequate finances and time due to the increased involvement of identifying, contacting, and building rapport with potential participants, as well as developing appropriate study materials [26].

#### Lack of research information and awareness for participants

A barrier to research participation was limited awareness of clinical trials and research opportunities among minority groups [17], for example about dementia research among South Asian communities [32] and of research and research opportunities in general [33]. Additionally, Spencer et al. [28] reported that ethnic minority communities may perceive research as irrelevant due to a lack of information. Several factors may explain this including explanatory models of illness that patients hold [18], and over generalisation or oversimplification of illnesses such as depression [18]. This may also be due to stereotyping people of ethnic minority as being less interested in research participation [4].

While informed consent and information sheets are important, sections on ‘what if something goes wrong’ has been reported to cause confusion and be wary of the research [31] Individuals may not understand the potential benefits of participation in research due to insufficient or generic information given at the outset, impacting both recruitment and retention rates [4, 29]. Lack of awareness also overlaps several other barriers, as it highlights the lack of research resources available to ethnic minority groups. Furthermore, lack of awareness is likely to feed into research stereotypes that ethnic minority groups hold, which can perpetuate lack of trust and general scepticism around research.

#### The influence of family on research participation

Family plays an important role in the decision-making process for research participation in ethnic minority communities. Family members may pose barriers to recruitment [18, 33]. Similarly, within British South Asian communities the decision to take part in a clinical trial may be a collective family decision rather than the individual which may be influenced by an array of factors such as perceived financial burden of participation [31] and/or caring for children or grandchildren [27, 36]. For these reasons, ethnic minority individuals may be less likely to participate in research due to the influence of their communities. This highlights the importance of working with a community when planning and recruiting for research studies, as this can help break stereotypes and misconceptions around research participation.

#### Bias from healthcare providers or researchers

Healthcare providers may have biases or stereotypes towards ethnic minority groups [4], leading to difficult relationships and further exacerbating the mistrust in research by potential participants. Studies suggest that healthcare providers have felt less confident in explaining trials to non-English speaking patients as they felt they had less interest in taking part in trials [4]. This however may be related to the lack of language barrier provisions made when explaining research non-English speaking patients, as they are unlikely to understand what research participation entails. Other myths include that some communities are ‘hard to reach’, displaying deviant behaviour, or be perceived as a ‘research risk’ [4, 37], perpetuating negative perceptions of some ethnic minority groups being difficult to access [18], which then results in mistrust of the researchers and reluctance to participate in clinical trials. This is important to address in the informed consent process, as a lack of trust can lead to refusal to take partake [4]. Furthermore, although this barrier is framed from the healthcare provider/researcher’s standpoint, this is likely to perpetuate several individual level barriers such as mistrust of healthcare professionals/researchers and lack of awareness. This highlights the need for intervention at researcher/healthcare professional level.

### Facilitators for engaging ethnic minority individuals in research

#### Adopting a personalised approach

Personalised approaches to recruitment are important [33]. Personal approaches by trusted community leaders and reliable social networks can improve recruitment. Personalised approaches are important as participants are seen both as an individual and part of the groups with which they identify [18, 26–28, 30, 32]. Examples of personalised approaches are taking the time to build rapport and relationships with participants, using culturally sensitive communication styles, personal phone calls rather than automated calls, paying attention to significant religious dates and providing participants with thank you letters [27, 28, 33]. As ethnic minority groups can often be sceptical of research participation due to a lack of trust in healthcare/research, taking the time to ensure recruitment approaches are suitable with the input of community leaders for the community will help with breaking these barriers and building trust.

#### Providing participants with culturally appropriate incentives

Providing culturally appropriate incentives such as covering travel costs has been identified as a facilitator for community-based recruitment as well as help with encouraging gatekeeper assistance with recruitment [29]. Culturally inappropriate incentives may hinder recruitment [18, 27, 29, 30, 32], therefore factoring in incentive planning at the early stages of research development is likely to support research recruitment.

#### The use of translators and linguistically appropriate study materials

To improve rapport building between researchers and participants, bilingual staff from ethnic minority groups to ensure research teams are diverse and visually representative of the population being recruited, and multilingual study materials are important [17, 18, 30, 32, 33]. Study materials should be appropriate for all literacy levels [17] which helps the participants feel appreciated and accommodated for. It also eases communication between researchers and potential participants [18].

Culturally appropriate communication strategies throughout the research process include researchers sending letters to potential participants prior to study contact, mass mailing, keeping phone calls short, reminder phone calls, regular study updates, appropriate readability of materials, the use of multimedia, and appointment cancellations followed up [33]. Keeping participants up to date with research outputs and educating communities on how outputs will benefit both them and the community is also beneficial and for participants to feel heard and valued throughout the research process. These factors are likely to work towards mitigating language barriers, cultural barriers, and work towards ensuring research is transparent and participants understand the benefits of the research they intend to participate in.

#### Adopting culturally sensitive research strategies

Community champions have been shown to be useful in study recruitment. Community champions are often key members of the community who are familiar with cultural norms and practices that exist and have connections with community groups. They can also ensure research is culturally appropriate, for example in some cultures matching the gender of the researcher with the participants should be respected [32]. Additionally, it is often assumed that South Asians are a homogenous sample, however there are several cultural differences between different sub-minorities of South Asians [32]. Hence, community champions should be able to recognise this and ensure they are able to avoid using language which could be appropriate for one sub-culture, however potentially offensive for another [32].

In addition, at the research planning stage, researchers should make efforts to make research accessible and tackle logistical barriers such as being flexible with participation timings and study location (including home based assessments if possible), advice on finding childcare, transport, and reducing costs associated with trial or study participation. This can help tackle socioeconomic and logistical challenges.

#### Providing researchers with support on cultural competency

To build trust and improve rates of recruitment, researchers should attend cultural competency training [29, 33] to ensure that the approach and work with the community is culturally appropriate [31]. The importance of culturally sensitive communication was highlighted across reviews, alongside importance of tailoring communication strategies to the cultural context of participants [4, 29, 31]. For example, in mental health research, culturally sensitive communication helps address stigma and misunderstandings about mental health conditions [31].

Researchers should be aware of religious and social commitments, especially when scheduling study participation [32]. Working collaboratively with community champions and family members where the family are likely to influence choice of participation is beneficial for family members [32] as is engaging and educating the family to build trust and confidence, and clarifying what participation would involve. Engaging with the community through multiple sources helps to overcome any stigma and mistrust associated with research [32]. Reciprocal mentoring with community champions would assist the research process.

#### Community engagement

Community engagement to increase ethnic minority engagement in research [17, 26, 32], includes the use of bilingual researchers to communicate with the community and reduce the chance of any misinterpretation of research information. Community based participatory research (CBPR) approaches significantly enhanced recruitment and retention [28]. These included using a community advisory board, community leaders, groups and organisations, and direct outreach to participants. The involvement of a culturally competent individual who is viewed as an ‘insider’ (community champion), is important in recruitment. Prior consultation with community members through a patient and public involvement (PPI) approach was also seen to be a beneficial strategy [17, 28, 32]. It is important to ensure gatekeepers are actively involved and aware of the inclusion criteria of research, informed throughout the recruitment process, and attend relevant cultural competency training to avoid stereotyping participants. Through active community engagement and collaborations with local communities, researchers can ensure research is culturally appropriate and accessible for the community.

#### Study advertising and educational workshops

The use of various community favoured social marketing recruitment channels, using culturally appropriate messages in local communities in collaboration and consultations with community champions and local gatekeepers was seen as a facilitator for research engagement. This could include education workshops set up for participants to ensure understanding the processes and the benefits of participation to them and their communities [4, 30, 33]. These should be planned and carried out at regular intervals of the research as patient and public involvement groups [32].

Bodicoat et al. [33] emphasised the role of social marketing campaigns tailored to specific ethnic communities, using culturally relevant messages and media channels, were successful in raising awareness about dementia and the importance of research participation. It is also important to consider the application of these methods across generations, as social marketing campaigns may be less appropriate for older generations. Liljas et al. [27] suggested that face to face gatekeeper referrals have been useful for ethnic minority groups, and recruitment is more likely to be successful if participants had heard about the study by word of mouth initially. For this reason, it is suggested that methods of engagement are combined in order to ensure research is accessible and research opportunities are promoted effectively in the community. Involving family in educational sessions with an opportunity for them to ask researchers questions would also be helpful as they are a significant contributing factor in decision making in ethnic minority families [18].

No policy level facilitators were identified, highlighting the lack of recommendations available on a policy level. These policy level gaps for engaging underrepresented ethnic minority groups in healthcare research is likely a complex issue rooted in various systemic, institutional and societal factors. This gap may exist for several reasons, and it is important to understand and acknowledge these barriers in order to address the issue and work towards creating more inclusive research environments.

The lack of policy level facilitators for research participation may be partly due to there being broad healthcare research inclusion policies, however there being a lack of specific, targeted policies to engage ethnic minority groups [38]. Policies may fail to prioritise the inclusion of ethnic minority groups in research due a lack of awareness of insufficient resources for research to focus on this area. Furthermore, healthcare institutions may lack the necessary resources and expertise to engage ethnic minority groups in research effectively. This can result in people of ethnic minority feeling misunderstood and disrespected when approached for research participation [4]. It is important to recognise the lack of policy level facilitators as addressing this gap would require efforts at policy level to create more inclusive, culturally competent and fair research environments.

## Discussion

The purpose of this umbrella review was to summarise the reviews exploring barriers and facilitators for the engagement of ethnic minority groups in research across various topics, including mental health and dementia research. Eleven studies were looked at in this umbrella review, and this is the first umbrella review to explore barriers and facilitators for ethnic minority research engagement in line with the socio-ecological framework.

Despite the diversity of research contexts, common themes emerge that influence participation among ethnic minority groups. This review identified several common barriers, such as mistrust of trust of healthcare and research in general [18, 26, 29], cultural and language differences that were not appropriately accommodated for in research [18, 27, 32], logistical challenges (some of which were related to socioeconomic status) [30, 33], and lack of awareness around research and research opportunities [28, 32].

Key facilitators included community engagement and PPI from the outset of research [28, 32], researchers adopting culturally appropriate approaches such as the recruitment of community champions and recruitment of diverse research teams to bridge the language and cultural gap between researchers and participants [31, 33], ensuring research is accessible on all communication levels i.e. literacy skills and linguistically [27, 29], practical support through networking with community groups [28], and providing potential participants and those close to them with educational research materials prior to research recruitment [28, 32]. Multi-faceted approaches which combine community engagement, culturally appropriate materials, and flexible study designs were found to be the most effective [29].

The mistrust of medical institutions among ethnic minority groups is deeply rooted in historical and ongoing discrimination [31]. Cultural and language differences further exacerbate this mistrust, making it challenging for researchers to communicate effectively with potential participants. Furthermore, community engagement and culturally tailored approaches are critical in building trust and overcoming cultural barriers, as it helps potential participants feel heard and valued. Practical support, such as providing transportation and flexible scheduling, directly addresses logistical challenges, while educational workshops around the nature of research can bridge the knowledge gap about the benefits of research participation. The main outcome from this was the need to address specific illness related stigma, both on a community and individual basis.

The findings of this umbrella review are consistent with previous reviews that highlight mistrust and logistical challenges as major barriers [38, 39]. The mistrust is pronounced among ethnic minority populations who have faced longstanding health disparities and exclusion from clinical trials [38]. Furthermore, George et al. [38] discussed how ethnic minority populations are often sceptical when it comes to research participation due to concerns surrounding privacy, exploitation, and lack of representation in the decision-making process. These factors can create barriers to engagement and encourage a reluctance to trust healthcare systems, reinforcing the need for strategies to address these challenges.

Additionally, cultural barriers emerged as a barrier in the current review. Scharff et al. [40] explored the role of cultural competency in research and highlighted that a lack of understanding and respect for cultural values within healthcare institutions can discourage ethnic minority populations from participating in research [40]. The study found that a lack of cultural diversity and competence among researchers contributes significantly to mistrust, as ethnic minority patients often receive less information about healthcare and research. Moreover, the present review encouraged the use of community based participatory research (CBPR). Research has shown CBPR to enhance trust and improve recruitment by actively involving community members in the research process [41]. This supports the need for a more inclusive, collaborative approach to research with ethnic minority groups. Research conducted by Wendler et al. [42] challenges the assumption that minority groups are less willing to participate in health research and discusses factors influencing their participation, including trust and perceived exploitation. This research highlights the needs for intervention at research level to ensure research is inclusive and accessible [42].

Research shows ethnic minorities, particularly those from lower socioeconomic backgrounds, often face difficulties related to time constraints, transportation, and lack of financial resources to participate in research [38]. This review found that offering culturally appropriate incentives, such as compensation that reflects the community’s needs, could act as a facilitator.

Facilitators identified in this review, such as community engagement, personalised approaches, and educational workshops, have been shown to increase recruitment and retention rates among ethnic minority populations in healthcare research. Community-based initiatives that involve ethnic minority groups in the research process have been particularly successful in building trust and addressing concerns [38].

The barriers and facilitators have been presented in line with the socio-economic framework which identifies factors on an intrapersonal, interpersonal, community and policy level. As illustrated in Fig. 2, majority of the barriers and facilitators identified in this umbrella review are at an intrapersonal and interpersonal level, with community level facilitators. No policy level facilitators were identified. This in itself could be interpreted as a barrier, as the lack of policy guidelines make changes difficult to implement. Future research on policy is required in order to inform recommendations to increase the engagement of ethnic minority groups in research.

There are several implications for practice that can be derived from this review. Within study protocols, time should be allocated to building rapport with community groups in order to ensure they are aware of the research, and they are familiar with researchers in order to build trust. This would also increase the awareness of research among the community. Researchers should invest in building long-term relationships with community organisations, provide cultural competency training for staff, and develop multilingual materials at the outset of research. Additionally, study teams should make an effort to recruit multilingual researchers where appropriate, as this breaks the language barrier and helps participants feel heard and understood.

Grants should include funding opportunities specifically aimed at supporting inclusive research practices and create guidelines for increasing diversity in research to design more inclusive research protocols and outreach programs that are sensitive to the needs of ethnic minority populations. Research funding should factor in additional costs associated with logistical challenges that can commonly arise such as lack of transport, lack of childcare, in order to mitigate these barriers and ensure the research is designed to be inclusive of all populations. Additionally, researchers should offer flexible scheduling and remote participation options for communities where logistical constrains present participation barriers. On a policy level, it is important to develop and fund policies that encourage collaboration between researchers and community groups. These collaborations can help ensure that research is transparent and culturally relevant. Involving community champions in the research design process, recruitment and research dissemination stages is a beneficial strategy to implement trust through collaboration and ensure research aligns with the community priorities. Additionally, researchers should engage in cultural competency training. This can help researchers understand the historical context that influences mistrust and ensure they approach research with ethnic minority communities appropriately. To ensure research is accessible, policy leaders should mandate the availability of multilingual research materials to ensure language barriers are not a factor for exclusion.

A strategy to increase awareness of the importance of research may be through health campaigns to raise awareness about research, the benefits of research, and how they can get involved. These should be developed in collaboration with community groups to ensure they are culturally appropriate and available in widely used media channels to disseminate information effectively. The strategies proposed can build trust, increase accessibility and increase awareness, leading to more inclusive healthcare research. Policy interventions should provide supportive frameworks to enable researchers to engage effectively with ethnic minority populations, ensuring diverse representation in the research process.

This umbrella review synthesises a wide range of studies across multiple research topics, providing a comprehensive overview of barriers and facilitators. A possible limitation is that the umbrella review may be subject to publication bias, as studies with negative findings are less likely to be published. Additionally, non-English studies have been excluded from this review, which means insights from other areas of the world could be missed. This was however done to ensure the specificity and relevance of the review for UK based researchers where the infrastructure of research and healthcare is unique compared to other parts of the world.

## Conclusions

This umbrella review highlights the complex interplay of barriers and facilitators that influence the participation of underrepresented ethnic minority populations in research. While barriers such as language, cultural stigma, mistrust of researchers and healthcare, and socioeconomic and logistical constraints are significant, they are not impossible to address. Effective strategies that include community engagement, culturally sensitive approaches, flexible research designs, and educational workshops for potential participants and their families can facilitate greater inclusion of ethnic minority populations in research. As discussed in this umbrella review, the challenges faced are complex and dependent on the research area, therefore although guidelines can be created, it is important to address barriers on an individual research topic in order to take a personalised approach. Addressing these barriers through a tailored and community-centred approach is essential for ensuring that research is representative and that the findings are applicable to diverse populations. Additionally, this review outlines the needs for systemic and policy level interventions to support and encourage the inclusion of ethnic minority groups in healthcare research. Future research should continue to explore the need for policy changes and recommendations for engaging underrepresented groups, with a focus on building trust and demonstrating the tangible benefits of research participation with the aim to making research inclusive for all ethnic minority groups. Future directions for policy development in the participation of ethnic minority populations in healthcare research should focus on community engagement, culturally tailored research resources, cultural competency training for researchers, and addressing socioeconomic barriers in the research planning stages in collaboration with community groups. This is to ensure research is inclusive for all ethnic groups. Future research should explore how policy changes can foster a sustainable, culturally sensitive and inclusive healthcare research environment, which will lead to equitable healthcare outcomes for ethnic minority groups.

## Data Availability

This is a review which includes publicly available peer reviewed published data. Details of individual research studies included is provided within the manuscript.

## Abbreviations

UK: United Kingdom
USA: United States of America
NIHR: National institute for Health & Care Research
BAME: Black, Asian & Minority Ethnic
NIH: National Institute of Health
JBI: Joanna Briggs Institute
CBPR: Community based participatory research
PPI: Patient and public involvement
